# Peripheral Blood Lymphocyte Subsets (CD4^+^, CD8^+^ T Cells, NK Cells) in Patients with Cardiovascular and Neurological Complications after Carotid Endarterectomy

**DOI:** 10.3390/ijms160510077

**Published:** 2015-05-04

**Authors:** Katarzyna Kotfis, Jowita Biernawska, Małgorzata Zegan-Barańska, Maciej Żukowski

**Affiliations:** Department of Anaesthesia, Intensive Care and Acute Poisoning, Pomeranian Medical University, Teaching Hospital No. 2, 70-111 Szczecin, Poland; E-Mails: lisienko@wp.pl (J.B.); mazegan@wp.pl (M.Z.-B.); zukowski@sci.pum.edu.pl (M.Z.)

**Keywords:** carotid endarterectomy, helper T lymphocytes (CD4^+^), cytotoxic T lymphocytes (CD8^+^), NK cells

## Abstract

Background: The aim of the study was to evaluate the differences in the circulating immune cells’ subgroups after the atherosclerotic plaque removal in patients presenting with postoperative complications as compared to the patients without complications after carotid endarterectomy (CEA). Methods: Patients with significant carotid atherosclerosis (*n* = 124, age range: 44 to 87 years) who underwent CEA were enrolled in a prospective study. The immunology study using flow cytometry was performed to determine the percentages of peripheral blood T cells (CD4^+^, CD8^+^, Treg—CD4^+^/CD25^+^) and NK (natural killer) cells before and after the procedure. The data were expressed as the percentage of total lymphocytes ± the standard error of mean. Results: The mean percentage of lymphocytes (61.54% ± 17.50% *vs.* 71.82% ± 9.68%, *p* = 0.030) and CD4 T lymphocytes (T helper, 38.13% ± 13.78% *vs.* 48.39% ± 10.24%, *p* = 0.027) was significantly lower six hours after CEA in patients with postoperative 30-day cardiovascular and neurological complications as compared to the group without complications. On the other hand the mean NK level in the group with complications was significantly higher (21.61% ± 9.00% *vs.* 15.80% ± 9.31%, *p* = 0.048). Conclusions: The results of this study suggest that after carotid endarterectomy the percentages of circulating immune cells subsets differ in patients with and without postoperative complications.

## 1. Introduction

Atherosclerosis is one of the most problematic medical conditions of the 21st century leading to cardiovascular and neurological complications. Cardiovascular disease in turn is the main cause of death worldwide, with great geographical diversity, accounting for 1.4 million deaths in developed countries to 5.7 million deaths in developing regions [1,2]. Atherosclerosis has been identified as a chronic, low grade inflammatory disease. It is the result of the activation of the innate immune system initiated by the damage of the endothelium and lipid accumulation in the vessel wall, with adaptive immunity deeply involved in this ongoing disease process of large and medium-sized arteries [3,4].

It has been shown in numerous studies that atherosclerotic plaques consist of necrotic cores, calcified regions, accumulated modified lipids, inflamed smooth muscle cells, endothelial cells, leukocytes, and foam cells [5]. The composition of the plaques indicates that atherosclerosis is a complex disease involving the vascular, metabolic, and immune systems [6].

The recognition of atherosclerosis as a chronic inflammatory disease with the key role being played by macrophages and T lymphocytes and the cytokines released by them, led to an understanding of disease dynamics in different patients and non-homogenous set of symptoms [7,8]. The course of the disease and the dynamics of changes within the atheroclerotic plaque greatly depend upon the type of T lymphocytes engaged in the process in the endothelium of the arteries.

The CD4^+^ and CD8^+^ T lymphocytes are types of leukocytes known as T cells with surface markers known as CD4 and CD8 respectively. The CD4^+^ cells are commonly known as T-helper (Th) cells as their role is to help to detect and fight off infections, mainly bacterial and viral. The CD8^+^ cells are known as cytotoxic T cells (T cytotoxic) and detect and participate in eliminating infections caused by viruses and diseases such as cancer [3].

It is mainly the increase in Th1 subtype lymphocyte activity that, if not counterbalanced by the regulatory mechanisms, leads to plaque instability and the occurrence of thrombotic episodes. There has been a number of published studies showing a similarity of the pathogenesis between atherosclerosis and autoimmune diseases, thus instigating the search for a specific triggering antigen.

One of the first antigens to be described was the oxy-LDL (oxygenated low density lipoprotein), which triggers clonal lymphocyte T activity within the atherosclerotic plaque. It has been shown that early atherosclerosis shows a Th1 response with predominant production of IgG2a antibodies against modified LDL, as well as IFN-γ and IL-6 [9].

Furthermore, detailed studies led to an identification of CD4^+^ cells to be the main effector cells within the carotid atheroclerotic plaques, with a special emphasis on T regulatory cells (CD4^+^CD25^+^), a small sub-population of lymphocytes decelerating the hyperactive immune action. The CD4^+^CD25^+^ cells belong to a large, non-homogenous group of T regulatory lymphocytes that perform their action through the action of IL-10 i TGF β anti-inflammatory cytokines. On the other hand, the role of CD8^+^ cells has not been fully elucidated in the pathology of the atherosclerotic plaque. They interact with viral antigens and are both cytotoxic and immunosupressive [10]. Tight control between Th1 and Th2 responses is achieved by Tregs, lymphocytes critical in maintaining immunological tolerance [11].

Another subset of lymphocytes which may also be important in atherosclerosis are the natural killer (NK) cells, which are innate lymphocytes that do not possess a T-cell receptor. It is becoming clear that these minor lymphocyte subsets may play an important role in regulating vascular immunity and the development of atheroclerotic lesions [12]. Although natural killer cells were isolated from early and advanced human atherosclerotic plaques, predominantly found in the shoulder regions, their role in atherosclerosis development is not entirely clear [6]. A loss- and gain-of-function study performed by Selathurai *et al.* provided definitive evidence that NK cells are atherogenic and their production of perforin and granzyme B is responsible for the expansion of necrotic cores [13].

Plaque rupture precedes the occurrence of symptoms in 50% of patients. The usual place for rupture is the fibrous cap within the plaque and is caused by macrophages and lymphocytes T infiltrating the cap itself. The macrophages release proteolytic enzymes, mainly collagenases, which leads to the destruction of the fibrous cap and intravascular thrombosis. The profile of pro-inflammatory cytokines isolated from the ulcerated atheromatous carotid plaques is typical for the cellular T lymphocyte immune response [14].

An acute decompensation of the cardiovascular system, including the cerebral circulation, presenting as stroke or transient ischaemic attack is caused by the instability of the atherosclerotic plaque secondary to the increased activity of the inflammatory cells [7]. The release of a range of pro-inflammatory cytokines, *i.e.*, TNF-α, IFN-γ, IL-1β, IL-6, enhances the inflammatory process caused by endothelial damage, concomitantly attenuating the reparatory processes [8,14,15]. The importance of the inflammatory processes, especially the disproportionate activation of particular populations of lymphocytes in the context of phenomena such as reperfusion and early restenosis is of particular importance. The majority of the available research papers regarding this issue are based either on animal studies or human studies within small populations [16]. Thus an observation in a larger human group seemed important and justified.

Furthermore, many studies have been performed with the analysis of lymphocyte subsets within the atherosclerotic plaques. What has not been investigated, however, is exactly how the population of circulating lymphocyte subsets behaves in patients presenting with cardiovascular and neurological complications after carotid endarterectomy in a 30-day observation. Thus the aim of the study was to detect the effects of carotid endarterectomy on peripheral blood lymphocyte subsets (helper and cytotoxic T lymphocytes—CD4^+^ and CD8^+^ T cells respectively, NK—natural killer cells), in patients with and without postoperative cardiovascular and neurological complications.

## 2. Results and Discussion

### 2.1. Results

#### 2.1.1. Demographic and Perioperative Data

Patients with significant carotid atherosclerosis (*n* = 124, age range: 44 to 87 years, 46 women—37% and 78 men—63%) who had undergone carotid endarterectomy (CEA) were enrolled in this study.

Patients in the group with early postoperative complications were older (73.80 ± 10.15 *vs.* 67.92 ± 8.97 years, *p* = 0.056), more frequently were men (90.00% *vs*. 60.53%, *p* = 0.064), with more episodes of dizziness prior to CEA (30.00% *vs.* 4.39%, *p* = 0.002). There were no other statistically significant differences between the two subgroups. The demographic data for the study group is depicted in Table 1.

**Table 1:** Demographic data and concomitant medications in groups with and without 30-day complications.

Data	Complications (*n* = 10) *n* (%)	No Complications (*n* = 114) *n* (%)	*p*
Age (years, *x̄* ± SD)	73.80 ± 10.15	67.92 ± 8.97	0.056
Male sex	9 (90.00)	69 (60.53)	0.064
ASA 1	0	0	0.205
ASA 2	8 (80.00)	68 (59.65)	
ASA 3	2 (20.00)	46 (40.35)	
ASA 4	0	0	
ASA 5	0	0	
BMI ( *x̄* ± SD)	26.65 ± 2.75	27.60 ± 4.06	0.416
Neurological episodes prior to CEA	8 (80.00)	67 (58.77)	0.188
RIND	4 (40.00)	51 (44.74)	0.772
TIA	3 (30.00)	13 (11.40)	0.093
Amaurosis fugax	1 (10.00)	7 (6.14)	0.634
Syncopy	1 (10.00)	7 (6.14)	0.634
Dizziness	3 (30.00)	5 (4.39)	0.002
Ischaemic heart disease	2 (20.00)	49 (42.98)	0.337
Diabetes mellitus	2 (20.00)	40 (35.09)	0.334
Acute myocardial infarction	1 (10.00)	20 (17.54)	0.542
Congestive heart failure	8 (80.00)	77 (67.54)	0.659
NYHA I	1 (10.00)	25 (21.93)	
NYHA II	1 (10.00)	12 (10.53)	
NYHA III	0	0	
NYHA IV	0	0	
Arterial hypertension	8 (80.00)	98 (85.96)	0.608
Dyslipidaemia	5 (50.00)	64 (56.14)	0.708
History of smoking	0	37 (32.46)	0.039
Use of ACE-I	7 (70.00)	75 (65.79)	0.787
Use of β-blockers	4 (40.00)	53 (46.90)	0.675
Use of statins	6 (60.00)	72 (63.16)	0.843

ASA—American Society of Anesthesiology; CEA—Carotid endarterectomy; RIND—Reversible ischaemic neurological deficit; BMI—Body mass index; SD—Standard deviation; NYHA—New York Heart Association classification; ACE-I—Angiotensin converting enzyme inhibitor; *p*—Statistical significance; *n*—number of patients.

Table 2 shows the intraoperative data for carotid endarterectomy procedures in groups with and without 30-day complications.

**Table 2:** Intraoperative data for carotid endarterectomy procedures in groups with and without 30-day complications.

Data	Complications (*n =* 10) *n* (%)	No Complications (*n =* 114) *n* (%)	*p*
Cross-clamping time (min, *x̄* ± SD)	20.57 ± 7.91	21.62 ± 7.18	0.661
Shunt	3 (30.00)	26 (23.21)	0.629
General anaesthesia (yes)	1 (10.00)	24 (21.05)	0.404
Ephedrine (use)	6 (60.00)	84 (73.68)	0.352
Ephedrine dose (mg, *x̄* ± SD)	11.00 ± 11.25	16.23 ± 15.84	0.347

SD—Standard deviation; min—Minutes; *p*—Statistical significance; *n*—number of patients.

#### 2.1.2. Postoperative Complications

Early complications as defined by the authors of this study occurred in 10 patients (8.06%) within 30 days after carotid endarterectomy. In seven cases, neurological complications were diagnosed (six cases of stroke and one case of a transient ischaemic attack). In three cases, cardiovascular complications occurred (1 × non-ST elevation myocardial infarction-NSTEMI, 1 × unstable angina-UAP, 1 × symptomatic sinus bradycardia and sinus arrest). None of these complications was present during the procedure or occurred immediately after the surgery.

Within that group, one death was observed on day 22 in the patient with transient ischemic attack (TIA) who suffered from respiratory and cardiovascular insufficiency after a cardiac arrest. Thus, a 30-day mortality rate was calculated as 0.8%. The complication rates according to the different criteria used in carotid endarterectomy research are shown in Table 3.

**Table 3:** Thirty-day complications in the study group (*n =* 124)—According to extended criteria used by the authors of this study and in published literature.

30-Day Postoperative Complications	%	*n*
Stroke/TIA + MI + UAP + Symptomatic bradycardia (Holter ecg) + death up to 30 days after CEA-according to criteria used by authors of the presented study	8.06	10/124
Stroke + MI + death ratio up to 30 days after CEA (SMDR)	6.03	8/124
Stroke + death ratio up to 30 days after CEA (SDR)	5.65	7/124
Death up to 30 days after CEA	0.80	1/124

TIA—transient ischaemic attack; MI—myocardial infarction; UAP—unstable angina pectoris; CEA—carotid endarterectomy; SMDR—stroke/MI/death ratio; SDR—stroke/death ratio; *n*—number of patients.

#### 2.1.3. Immunological Data for Patients with Early Complications

The comparison of peripheral blood cells subgroups based on flow cytometry is shown in Table 4. Prior to the CEA procedure the percentage of peripheral blood cells subset showed no statistically significant difference between groups with and without complications. In the group of patients with early postoperative complications a trend was noted towards a lower level of T helper lymphocytes (CD3^+^CD4^+^) prior to CEA (36.70% ± 12.07% *vs.* 44.38% ± 10.64%, *p =* 0.069), thus a lower CD4^+^/CD8^+^ ratio (1.31 ± 0.43 *vs.* 1.93 ± 0.92, *p =* 0.036).

**Table 4:** Peripheral blood cells subgroups analysis prior to carotid endarterectomy (CEA) procedure and 6 h post CEA for group with postoperative 30-days complications and without complications.

Peripheral Blood Cells (%, *x̄* ± SD)	Prior to CEA		*p*	6 h after CEA		*p*
	Complications *n* = 10	No Complications *n* = 114		Complications *n* = 10	No Complications *n* = 114	
Lymphocytes-total	26.09 ± 12.15	30.15 ± 10.63	0.339	15.32 ± 11.37	20.01 ± 8.92	0.134
Lymphocytes B	15.70 ± 14.55	11.21 ± 5.77	0.528	16.86 ± 15.65	12.49 ± 6.68	0.739
Lymphocytes T	66.14 ± 13.88	70.21 ± 10.14	0.440	61.54 ± 17.50	71.82 ± 9.68	0.030
NK Cells	18.17 ± 8.63	18.84 ± 9.28	0.951	21.61 ± 9.00	15.80 ± 9.31	0.048
Monocytes	6.68 ± 2.17	5.46 ± 1.72	0.070	5.01 ± 1.42	5.36 ± 1.68	0.588
Lymph.T helper	36.70 ± 12.07	44.38 ± 10.64	0.069	38.13 ± 13.78	48.39 ± 10.24	0.027
Lymph.T cytotoxic	29.20 ± 7.88	26.16 ± 8.57	0.183	24.13 ± 8.66	23.99 ± 8.12	0.827
%CD4^+^/%CD8^+^	1.31 ± 0.43	1.93 ± 0.92	0.036	1.67 ± 0.72	2.34 ± 1.16	0.122
Lymph. Treg	4.92 ± 2.34	5.55 ± 2.68	0.574	7.08 ± 1.47	6.14 ± 3.36	0.249

Lymph. T helper—CD3^+^CD4^+^; Lymph. T cytotoxic—CD3^+^CD8^+^; Lymph. Treg—CD4^+^CD25^+^; NK—natural killer; *p*—Statistical significance; *n*—number of patients.

The mean percentage of T lymphocytes (61.54% ± 17.50% *vs.* 71.82% ± 9.68%, *p =* 0.030) and CD4^+^ T lymphocytes (T helper, 38.13% ± 13.78% *vs.* 48.39% ± 10.24%, *p =* 0.027) was significantly lower six hours after CEA in patients with postoperative 30-day cardiovascular complications as compared to the group without complications. On the other hand the mean NK level in the group with complications was significantly higher (21.61% ± 9.00% *vs.* 15.80% ± 9.31%, *p =* 0.048).

### 2.2. Discussion

Based on both human and animal studies, atherosclerosis represents a chronic inflammatory process based on the innate and acquired activity of the immune system [3,10,17]. The working hypothesis for this study was to show a difference in the circulating immune cells’ subgroups after the atherosclerotic plaque removal in patients later presenting with postoperative complications as compared to the patients without complications. The activation of the inflammatory response may appear rapidly after the triggering stimulation. Animal studies suggest that in the first 24 h after a surgical stimulation a rapid increase in NK activity is visible, which is followed by a gradual decrease to baseline after eight days [18]. Studies performed by Profumo *et al.* analyzed the immune cell counts at 1, 3 and 6 months after the plaque removal [7,8]. The authors of this study decided to analyze the immediate postoperative behavior of the immune cells, as at the time when the study was designed no information was available to answer that question. There is a constant search for either preoperative or early postoperative factors that could answer the question of how the patient will do after the operation. Therefore the six hour time-point was chosen arbitrarily, as that was the time just after patient stabilization on the postoperative ward.

The first step was to define the term “postoperative complications”. In the available literature that term is rather narrow and covers either stroke/myocardial infarction (MI)/death ratio (SMDR) or stroke/death ratio (SDR). The authors of this study went a step further and applied additional postoperative monitoring to identify any cardiovascular deficit with sequential cardiac enzymes levels and Holter electrocardiography (Holter ecg) monitoring 24 h post-operatively. The broad definitions used by the authors resulted in a reported 10 cases (8.06%, 10/124) of 30-day complications which apart from death, stroke, TIA and MI also included an episode of unstable angina pectoris and symptomatic sinus bradycardia with transient sinus arrest picked up by the Holter ecg monitoring. When using the narrow criteria, the stroke/MI/death ratio was 6.03% (8/124) and the stroke/death ratio was 5.65% (7/124), thus the results were close to those recommended by the experts [19,20,21]. The international guidelines clearly state that the carotid endarterectomy procedures for symptomatic patients should carry a risk of postoperative stroke or death of less than 6%. In our study the mortality rate of 0.8% was also below the recommended 1% mortality set by the guidelines and by large randomized trials.

Moreover, none of these complications occurred during or immediately after the procedure. The blood for the immunology study was obtained six hours after the endarterectomy, *i.e.*, prior to the occurrence of any of the postoperative complications. Majority of the complications occurred within the first three days after the operation.

The innate immune response based on the activity of the macrophages, neutrophils, as well as the NK cells, presents as an important, yet not fully elucidated part of the atherosclerotic process. According to Whittman, the lack of those cell populations reduces the degree of the atherosclerosis [22,23]. Our study showed no differences in the percentage of NK cells prior to the endarterectomy procedure between the group with and without postoperative complications (18.17% ± 8.63% *vs.* 18.84% ± 9.28%, *p =* 0.951). Contrary to the preoperative status, the postoperative observation showed a higher percentage of circulating NK cells in the group with 30-day cardiovascular or neurological complications (21.61% ± 9.00% *vs.* 15.80% ± 9.31%, *p =* 0.048). This observation may show the prognostic effect of the increase in the percentage of the NK cells in relation to postoperative complications. This prognostic effect of the NK cells’ increase may be due to the reaction of the innate immunity to the surgical stress on the plaque imposed by eversion endarterectomy. The authors have found no other studies evaluating the NK cells’ level after endarterectomy. According to Clerc *et al.* patients with severe atherosclerosis have higher levels of circulating NK cells prior to revascularization. Similarly, Bruunsgard *et al.* evaluated the level of circulating NK cells to be higher in elderly patients with atherosclerosis, however the cytotoxicity of the NK cells was decreased [17,24,25].

It is possible that the complications occurred in those patients in whom the innate inflammatory reaction was induced with surgical manipulation on the atherosclerotic plaque. On the other hand the NK increase may be regarded as a marker of ongoing generalized inflammation triggering a cardiovascular or neurological outcome. The observation regarding the increase of NK cells in patients with early postoperative complications encourages further detailed studies in a prospective study within a larger group of patients as the main criticism of our study is the small size of the study group.

In atherosclerosis the inflammatory process occurs due to the activation of T lymphocytes both within the plaque as well as in the peripheral blood [26]. Evidence exists that circulating lymphocytes T CD4^+^ undergo a preferential activation towards Th1 cells when the plaque transforms from “silent” to “active”. This instability of the atheromatous plaque may be related to the predominance of T cytotoxic cells (CD8^+^) and the change in CD4^+^ to CD8^+^ ratio, which can cause the rupture of the plaque clinically visible as stroke, TIA or amaurosis fugax [27,28].

In our study a comparison of the percentage of lymphocytes in peripheral blood prior to the endarterectomy showed no difference when comparing the patients who were symptomatic prior to CEA to those who showed no symptoms preoperatively. On the other hand a comparison of patients presenting with complications within the first 30 days after CEA revealed a trend towards lower levels of T helper lymphocytes (CD3^+^CD4^+^) and a lower CD4^+^/CD8^+^ ratio.

The decrease of circulating T helper lymphocytes may lie in the fact that this sub-population of immune cells accumulates in the atherosclerotic plaques in different sites throughout the vascular system. There are many studies both experimental and clinical which showed that the majority of lymphocytes T present in the atherosclerotic plaque fall into the T helper category [29,30]. The key role of T helper lymphocytes further leads to a division into pro-atherosclerotic Th1 and anti-atherosclerotic Th2 lymphocytes [31]. In contrast to the above findings, research performed by Profumo *et al.* showed that there is no correlation between the phenotype of circulating T cells and the degree of atherosclerosis within the carotid artery. According to this group it is the cytokines TNF-α, IFN-γ and IL-4 which are known as markers of progressive atherosclerosis and that the shifts in the peripheral T lymphocyte sub-population do not show any correlation with the severity of atherosclerosis [8].

In the recent years reports regarding the role of naturally occurring T regulatory lymphocytes (CD4^+^CD25^+^) have emerged [32,33,34]. It has been shown that the lack of T regulatory CD4^+^CD25^+^ lymphocytes accelerate the formation of the atherosclerotic plaque in experimental modes [35,36]. Moreover, a study performed by Han *et al.* revealed that in patients with ischaemic heart disease the decrease in number of Treg lymphocytes (CD4^+^CD25^+^) led to an instability of the atherosclerotic plaque after an activation [26].

In our study, the percentage of T regulatory CD4^+^CD25^+^ lymphocytes showed no difference in the group with and without postoperative complications neither prior to the operation nor six hours afterwards. This result does not indicate a lack of response by the immune system to the surgical intervention on the atherosclerotic plaque. This change may depend upon the redistribution of the cell population between blood and infected tissues, which leads to an accumulation of lymphocytes CD4^+^CD25^+^ to suppress local inflammatory response [37]. The time-point for the Treg increase may be impossible to capture when single samples of blood are drawn. When critically analyzing the design of our study after performing it, we should have drawn more blood samples, including the period immediately after the surgical insult. The Treg lymphocytes should be analyzed in further studies in the context of new molecular markers and cytokine expression identifying the degree of atherosclerotic process in carotid arteries.

When analyzing the cardiovascular-related morbidity and mortality in patients with symptomatic atherosclerosis, factors other than an ongoing inflammatory process must be taken into account. Cardiac and vascular aging, modulated by certain metabolic pathways must be appreciated. Recently, it has been shown that calstabin2 protein and the activation of the AKT/mTOR pathway may play an important role in cardiac aging influencing the long term outcome in the elderly population [38]. Moreover, patients suffering from cardiovascular diseases have a specific pattern for circulating microRNA, regarded as regulators of gene expression. Many studies underline the role of microRNAs in the initiation and progression of the cardiovascular disease [39,40]. It has been shown recently that the use a miRNA-based strategy may enhance arterial repair [41].

The obvious limitations of our study, including a single center, observational activity, with limited external validity must be acknowledged. The time-points for the inflammatory cells’ analysis were set arbitrarily by the authors at 0 and 6 h. It remains a matter of discussion if the analysis should have taken place at 0, 6, 24 h and maybe 30 days after the procedure. Furthermore, it may be argued that the rate of complications reported by us is relatively high at 8.06%, but that is caused by the very broad definitions used by the authors.

The potential relationship between postoperative complications and inflammation, as indicated by the increase of NK cells shown in our study, must be analyzed with caution. During the carotid endarterectomy the unstable plaque has been removed totally. Therefore the inflammatory process induced by the unstable atheroclerotic plaque should decrease gradually. This was not the case in those patients in whom postoperative complications occurred, suggesting an ongoing generalized inflammatory response unrelated to the removal of the plaque or the surgical insult. The increase of the NK cells level may be a marker of a new unstable atherosclerotic site in patients with existing atherosclerosis, yet undiagnosed and clinically silent, but leading to complications later in the postoperative course. Similarly, the decrease of circulating T helper lymphocytes count may lie in the fact that these sub-populations of immune cells accumulate in the atherosclerotic plaques elsewhere in the cardiovascular system and may be an early marker of ongoing atherosclerotic process in the patient.

## 3. Experimental Section

### 3.1. Material and Methods

The prospective, observational study was performed in the Department of Anaesthesia and Intensive Care and the Department of Vascular Surgery of the Pomeranian Medical University. The institutional Bioethics Committee approved the study (BN-001/52/08, 27 May 2008). Informed consent has been obtained from each subject. Patients with significant carotid artery stenosis (60%–99% on ultrasound), both symptomatic and asymptomatic according to Ttrial of ORG 10172 in acute stroke treatment (TOAST) criteria for central nervous system ischaemia who qualified for planned carotid endarterectomy (CEA) procedure were included in the study [42].

We excluded patients who needed an emergency surgery, after a previous endarterectomy on either side or who had undergone any surgical procedure within the last six months. The list of exclusions covered any inflammatory or autoimmune diseases, as well as the use of any anti-inflammatory medications.

Pre-operatively, a full history was taken from all patients (family history, weight and height, body mass index (BMI), age, history of smoking, alcohol consumption, present medical and medication history). The results of their preoperative and postoperative sequential laboratory tests included: complete blood count (CBC), blood glucose, serum creatinine, ions (Na^+^, K^+^), clotting factors and cardiac enzymes. To enhance patient safety and to increase the amount of cardiovascular parameters obtained during the carotid endarterectomy procedure continuous Holter ecg monitoring with Suprima type 300-7 (Oxford, UK) was initiated prior to the procedure and continued for 24 h afterwards.

The surgical procedure was performed under regional anesthesia (superficial and deep cervical plexus block) with 0.5% bupivacaine (2 mg/kg) and 2% lignocaine (4 mg/kg). The operative technique was eversion endarterectomy. If after artery cross-clamping a deterioration of consciousness level occurred a shunt was implemented under general anaesthesia with sevoflurane and fractionated fentanil maintenance.

Post-operatively, full monitoring was performed in the recovery room and in the postoperative area of the vascular surgery ward for 24 h thereafter. Analgesia was achieved with the use of intravenous (iv) paracetamol and iv tramadol, occasionally with iv morphine.

#### Postoperative Complications Definitions

Early postoperative complications included any neurological or cardiovascular episode that occurred within the first 30 days after the procedure.

The neurological complications were diagnosed by a consultant neurologist and radiology studies and included: transient ischaemic attack (TIA), reversible ischaemic neurological deficit (RIND), amaurosis fugax and ischaemic stroke.

The cardiovascular complications included: cardiac arrest, acute coronary syndrome according to Global Registry of Acute Coronary Events (GRACE) criteria, unstable angina, arrhythmias and/or conduction deficits, exacerbation of chronic heart failure, acute heart failure. The cardiovascular complications were analyzed based on: clinical evaluation, 12-lead ecg, Holter ecg monitoring, as well as laboratory studies: troponin I, CK (creatinine kinase) and CK-MB (creatinine kinase isoenzyme, MB) evaluated 6 and 18 h after the procedure.

The surgical complications, *i.e.*, haematoma or restenosis requiring re-operation were also monitored. Patients requiring a re-operation were excluded from the analysis.

### 3.2. Immune Cell Preparation and Flow Cytometry Analysis

Whole blood (2.5 mL) was collected from each patient prior to CEA and 6 h after the procedure into sterile heparinized tubes with EDTA for total lymphocyte count and allowed determination of absolute counts. The blood drawn into EDTA tubes underwent the analysis of the following cells subgroups: lymphocyte B—CD3^−^/CD19^+^, lymphocyte T—CD3^+^, lymphocyte T helper—CD3^+^/CD4^+^; lymphocyte T cytotoxic—CD3^+^/CD8^+^, NK cells—CD3^+^/CD16^+^CD56^+^, CD4^+^ to CD8^+^ ratio, lymphocyte Treg—CD4^+^/CD25^+^. All antibodies were prepared according to the manufacturer’s directions and then were incubated with whole blood for 30 min at room temperature before red cell lysis and fixation using 1% formalin. The evaluation was performed using two-color immunofluorescence flow cytometry method with monoclonal antibody (MAb) panel (Simultest-BD, San Jose, CA, USA) for the desired cell surface proteins including fluorescein isothiocyanate (FITC) and phycoerythrin (PE)-conjugated Mab. Analysis was performed using flow cytometry (FACS Calibur-Becton Dickinson, San Jose, CA USA) with BD CellQuest software (version 2.0, system OS2, Becton Dickinson, San Jose, CA, USA).

The data were expressed as the percentage of total lymphocytes ± the standard error of the mean. The number of the studied CD4^+^CD25^+^ lymphocytes was expressed as a percentage of CD4^+^ lymphocytes.

**Figure 1:**
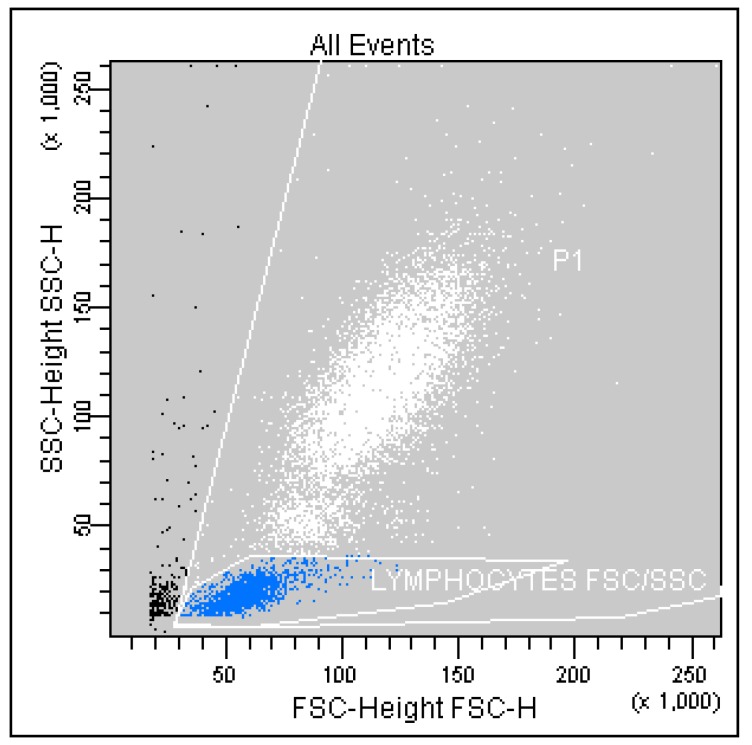
Gated lymphocyte sub-population based on SSC (Side-Scattered light) *vs.* FSC (Forward-Scattered light) measurement.

**Figure 2:**
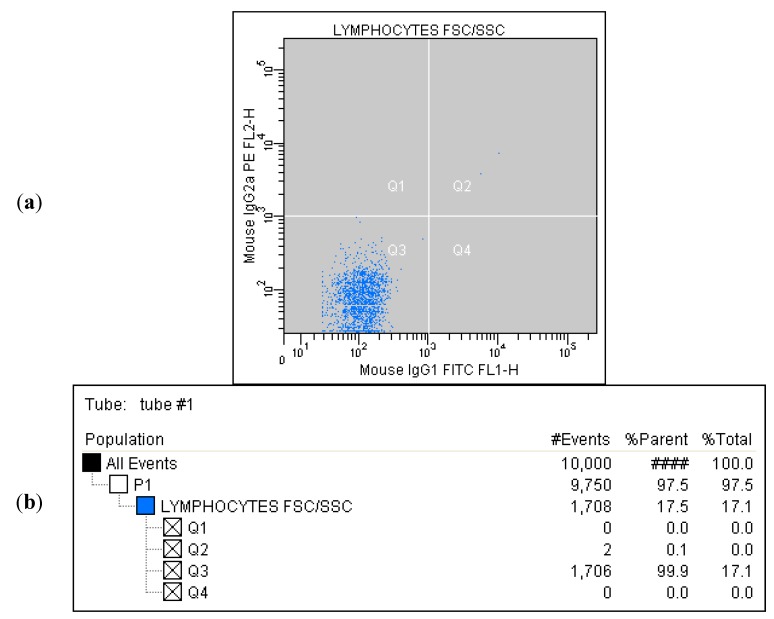
(**a**) Tube No. 1; Dot plot of MouseIgG1 fluorescein isothiocyanate (FITC)/MouseIg2a phycoerythrin (PE) with quadrant markers, showing negative control; (**b**) Tube No. 1; Gating tree showing gating strategy for Fluorescence-activated cell sorting (FACS) analysis as well as parent and total cell percentages within each of the gates for negative control.

**Figure 3:**
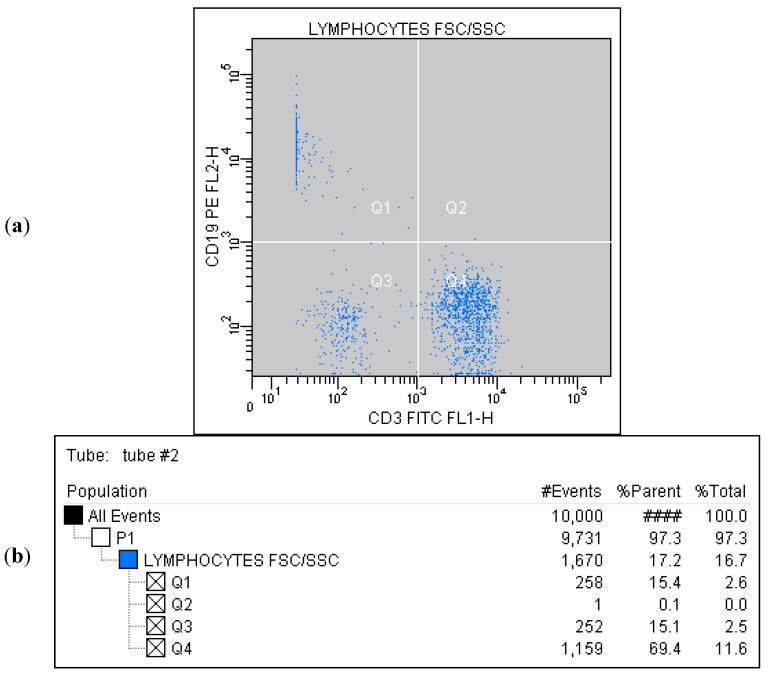
(**a**) Tube No. 2; Dot plot of CD3 FITC/CD19 PE with quadrant markers, differentiating lymphocytes B (CD19^+^) from lymphocytes T (CD3^+^); (**b**) Tube No. 2; Gating tree showing gating strategy for FACS analysis as well as parent and total cell percentages within each of the gates for CD19^+^ and CD3^+^.

**Figure 4:**
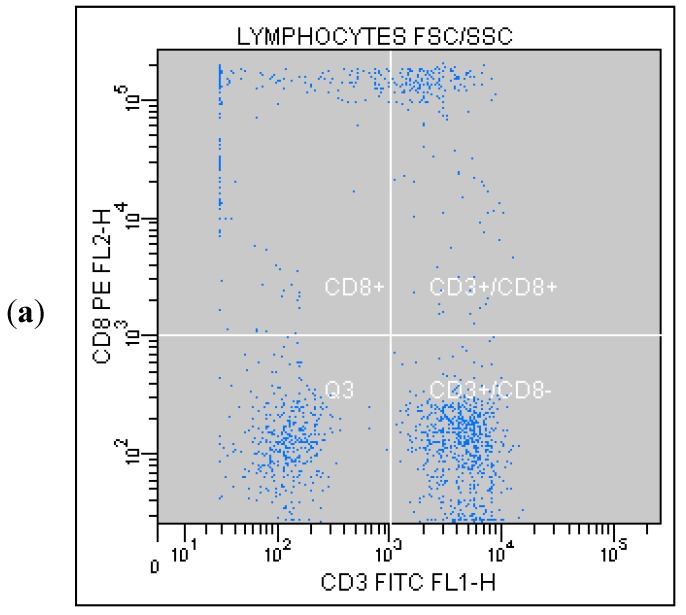

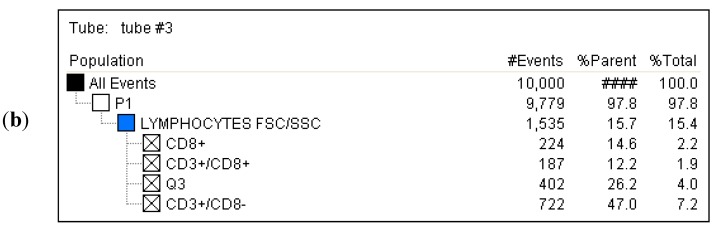
(**a**) Tube No. 3; Dot plot of CD3 FITC/CD8 PE with quadrant markers, differentiating lymphocytes T cytotoxic (CD3^+^/CD8^+^) from all lymphocytes; (**b**) Tube No. 3; Gating tree showing gating strategy for FACS analysis as well as parent and total cell percentages within each of the gates for CD8^+^ and CD3^+^.

**Figure 5:**
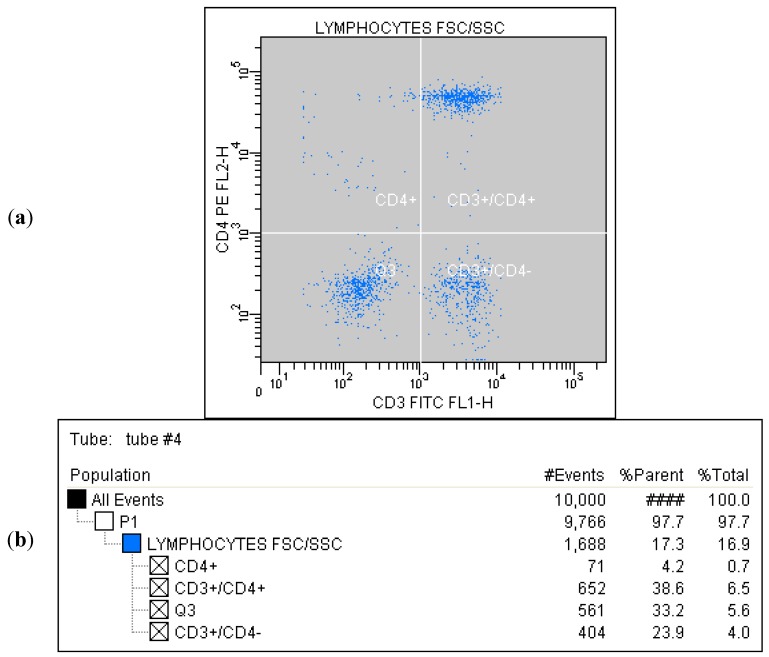
(**a**) Tube No. 4; Dot plot of CD3 FITC/CD4 PE with quadrant markers, differentiating lymphocytes T helper (CD3^+^/CD4^+^) from all lymphocytes; (**b**) Tube No. 4; Gating tree showing gating strategy for FACS analysis as well as parent and total cell percentages within each of the gates for CD4^+^ and CD3^+^.

**Figure 6:**
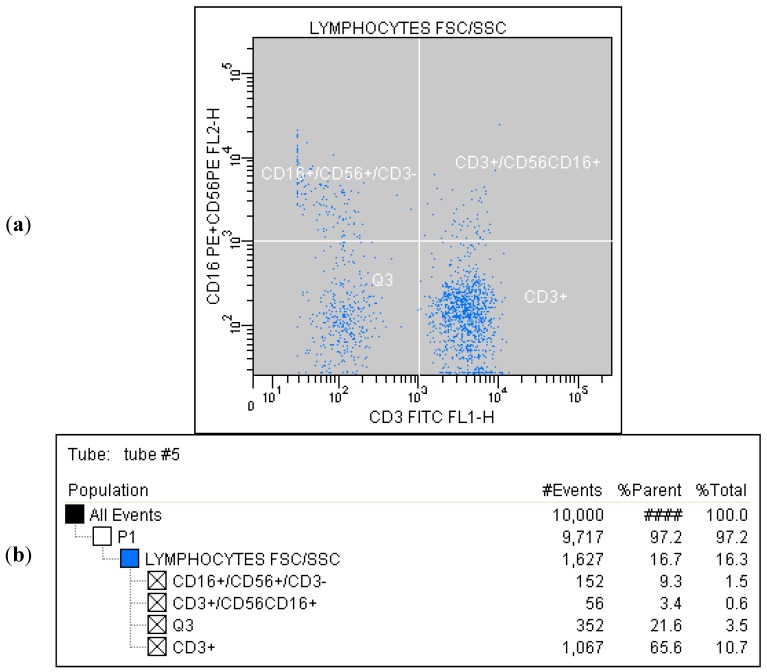
(**a**) Tube No. 5; Dot plot of CD3 FITC/CD16CD56 PE with quadrant markers, differentiating NK cells (CD3^+^/CD56CD16^+^) from all lymphocytes; (**b**) Tube No. 5; Gating tree showing gating strategy for FACS analysis as well as parent and total cell percentages within each of the gates for CD56CD16^+^ and CD3^+^.

**Figure 7:**
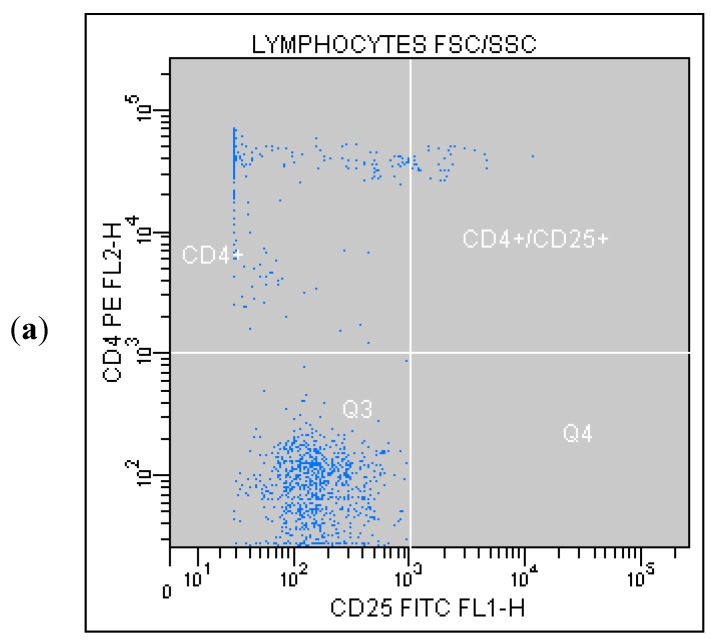

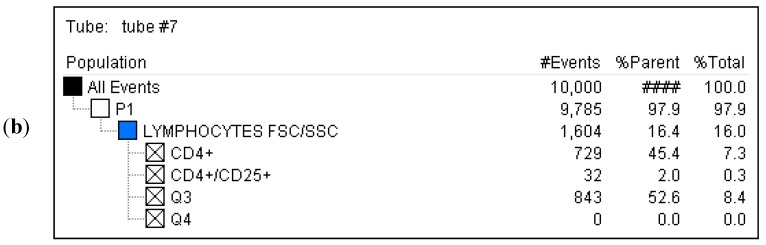
(**a**) Tube No. 7; Dot plot of CD25 FITC/CD4 PE with quadrant markers, showing Treg CD25^+^/CD4^+^ lymphocytes; (**b**) Tube No. 7; Gating tree showing gating strategy for FACS analysis as well as parent and total cell percentages within each of the gates for a representative CD4^+^CD25^+^ lymphocytes.

Correlated measurement of Side-Scattered light (SSC) and Forward-Scattered light (FSC) was used to differentiate the lymphocyte sub-population from the lysed whole blood cells. (Figure 1) The details regarding gating for different cells’ sub-populations are showed in Figure 2, Figure 3, Figure 4, Figure 5, Figure 6 and Figure 7.

### 3.3. Statistical Analysis

The aim of the study was to evaluate the differences in the circulating immune cells’ subgroups after the atherosclerotic plaque removal in patients later presenting with postoperative complications as compared to the patients without complications.

All continuous variables were checked for normal distribution using Kolmogorov-Smirnov test. These variables were expressed as mean and standard deviation. Statistical analysis between any two groups was checked using *t*-Student and Mann–Whitney test. For many groups, either variance analysis test was used (ANOVA) or co-variance test (ANCOVA), or Kruskal–Wallis test. To find statistical differences of the same subjects at different times *t*-Student test was used for dependent variables. The evaluation of statistical relationship between discrete variables was performed with either χ^2^ test or Fisher’s exact test. The value of *p* < 0.05 was considered statistically significant. Statistical calculations were made using STATA 11 (No. 30110532736, StataCorp LP, College Station, TX, USA) program.

## 4. Conclusions

Based on our study, after the removal of atherosclerotic plaque a statistically significant reduction in the percentage of CD4^+^ T helper cells and an increase in the NK cells level occurred in patients who later experienced postoperative complications. The relationship between the immunology response and the clinical picture remains unclear and warrants further studies on a larger group with atherosclerosis undergoing carotid endarterectomy.
